# Author Correction: Seroepidemiological study of factors affecting anti-spike IgG antibody titers after a two-dose mRNA COVID-19 vaccination in 3744 healthy Japanese volunteers

**DOI:** 10.1038/s41598-023-28884-7

**Published:** 2023-02-02

**Authors:** Aya Sugiyama, Akemi Kurisu, Shintaro Nagashima, Kiyomi Hando, Khilola Saipova, Sayyora Akhmedova, Kanon Abe, Hirohito Imada, Md Razeen Ashraf Hussain, Serge Ouoba, Bunthen E, Ko Ko, Tomoyuki Akita, Shinichi Yamazaki, Michiya Yokozaki, Junko Tanaka

**Affiliations:** 1 Department of Epidemiology, Infectious Disease Control and Prevention, Graduate School of Biomedical and Health Sciences, Hiroshima University, 1-2-3, Kasumi, Minami-ku, Hiroshima, 734-8551 Japan; 2 Department of Clinical Radiology and Oncology, Andijan State Medical Institute, Andijan, Uzbekistan; 3 Department of Cardiorheumatology, Republican Specialized Scientific-Practical Medical Center of Pediatrics, Tashkent, Uzbekistan; 4 Unité de Recherche Clinique de Nanoro (URCN), Institut de Recherche en Science de La Santé (IRSS), Nanoro, Burkina Faso; 5 Payment Certification Agency (PCA), Ministry of Health, Phnom Penh, Cambodia; 6 Division of Clinical Laboratory Medicine, Hiroshima University Hospital, Hiroshima, Japan

Correction to: *Scientific Reports* https://doi.org/10.1038/s41598-022-20747-x, published online 29 September 2022

The Supplementary Information 2 file published with this Article contained an error in Supplementary Figue 2 (D) where the antibody titers for BNT162b2 and mRNA-1273 were reversed. The original Supplementary Information 2 file is provided below.

This error has now been corrected in the Supplementary Information 2 file that accompanies the original Article.

## Supplementary Information

Supplementary Information 2.

